# A research agenda to promote affordable and quality assured medicines

**DOI:** 10.1186/2052-3211-7-2

**Published:** 2014-03-13

**Authors:** Warren A Kaplan, Veronika J Wirtz

**Affiliations:** 1 Department of Global Health, Boston University School of Public Health, Boston, USA

## Abstract

Promoting generic medicines to increase access to essential medicines is relevant to achieve the Millennium Development Goal (MDGs) and post 2015 goals.

There are several barriers to encouraging wider use of generic medicines in health systems, e.g. the widely-held perception that low price equals low quality and misalignment of provider and consumer incentives. Overcoming the complex barriers and other challenges can be re-formulated as a ‘generic medicine evidence-based policy agenda’: (1) What policy and strategies can increase consumer trust in the quality of all medicines granted market authority including generic products? (2) Are there differences in prices between branded and unbranded generics? (3) What are synergies between policies that can enhance promoting of generic medicines effectively?

Evaluating the policies promoting generic medicines will be critical to create evidence that countries can use to implement policies in their local settings.

## Background

One third of the population does not have access to essential medicines and promoting generic medicines to increase access to essential medicines is relevant to achieve the Millennium Development Goal (MDGs) and post 2015 goals [1]. Generic medicines are generally less expensive than originators. However, they offer the same efficacy, safety and quality as the originator. For the purposes of market approval, the interchangeability of a generic medicine and the corresponding original (i.e., ‘innovator’) product is based on criteria which require that the generic product have the same amount and type of active pharmaceutical ingredient and the same therapeutic effectiveness as the original product. One of the reasons that generics are priced lower than originators is that medicines regulatory authorities do not ask generic manufacturers to repeat the clinical trials of the originator in order to grant market authorization.

For many important compounds (e.g., including Lipitor® (atorvastatin; Pfizer), Plavix® (clopidogrel; Sanofi–Aventis/Bristol-Myers Squibb) and Zyprexa ®(olanzapine; Eli Lilly & Company)) —patents have already expired or will expire soon and making them more affordable for many payers, including third party payers such as insurance schemes. All of these medicines are used to treat non-communicable diseases which are already responsible for the majority of deaths worldwide.

Unfortunately, there are several barriers to encouraging wider use of generic medicines in health systems. These barriers are complex and may not be easy to overcome. One of the most intractable barriers is the intellectual property/access to medicines narrative, with complex issues involving the link between multinational pharmaceutical company patents and prices, delay of generic medicine approvals (including "pay for delay"), Trade related aspects of Intellectual Property Rights (TRIPS), Free Trade Agreements, absence of large insurance schemes promoting generic medicine use, incompetent medicines regulatory authorities and the like.

One important barrier is perceptual. In a 2008 experiment, 82 men and women were asked to rate the pain caused by electric shocks applied to their wrist, before and after taking a pill. Half the participants read that the pill was $2.50 per dose- the other half read that it had been discounted to 10 cents. All pills were placebos. The pills had a strong placebo effect in both groups but over 80% of those using the expensive pills reported significant pain relief, compared with around 60% on the cheaper pills [2]. The fact that the strength of a placebo-derived response was greater when the medicine was perceived as more expensive suggests a major psychological barrier to increased use of generic medicines, “if it’s expensive, it must be better” or its inverse, “if it’s cheaper, it can’t be good.”

### Overcoming these, and other, challenges can be re-formulated as a ‘generic medicine evidence-based policy agenda’

1. Increasing consumer trust in the quality of all approved medicines including generic products

Generic medicines policies include those to promote competition in the pharmaceutical sector such as providing rapid market authorization, providing clinical data for generic medicine companies prior to patent expiry, and mandated generic prescription or dispensing. Each of these policies however, depends on a perception, which must be based in reality that the generic product is of assured quality. If generics do not have such credibility, prescribers and end users will prefer to use the originator product. Overcoming the perception of “cheaper is not as good” is possible via public information campaigns and active promotion by insurance schemes. However, there is insufficient information on how much public information campaigns actually change perception, particularly for low and middle income countries (LMIC). There is also a scarcity of information on the impact of insurance schemes’ promotion of generic medicines to beneficiaries in other than high-income settings.

Further, through increasing transparency of medicine quality testing, it has been shown that low price is not equated with low quality. However, there is a lack of studies in low- resource settings. What are the best practices of functioning and reliable Medicine Regulatory Authorities (MRA) to increase consumer trust in the quality of all approved medicines including generic products? What are the best practices of other stakeholders such as physicians and pharmacies to increase consumer trust in the quality of approved medicines including generics? It appears the evidence is lacking to answer these questions. Policy decisions about changing stakeholders’ perceptions of medicines quality should be based on comprehensive evaluations.

2. The “branded generic” conundrum

Branded generics are generic medicines that are sold under a brand name instead of the non-proprietary name (e.g. Nurofen® as a brand name instead of ibuprofen, the non-proprietary name). Many persons will go to a private retailer, possibly driven by the same perceptions discussed above. A recent study has shown that the dominant form of generic consumed in the private sector of many LMICs is branded generics [3]. However, there is little comprehensive information on how prices of unbranded generic medicines compare with branded generic medicines. Research by WHO and Health Action International have shown that branded generics can often more expensive than INN generics [4]. Is this context specific? Is it generally true that branded generic medicines are more expensive than unbranded medicines? Further analyses of the price, availability and affordability of branded generics versus unbranded generics are critical to illuminate these issues.

3. Synergies between stakeholders and policies

Yadav, Sekhri and Curtis [5] looked at pharmaceutical supply chains and noted that the incentives of stakeholders to perform certain actions (e.g., forecasting demand, raising or lowering prices) are often misaligned. They believed that misaligned incentives create effects such as over-reactions, unnecessary interventions, second guessing, mistrust, and distorted information; all of which can degrade the ability of a supply chain to match supply and demand.

One could, in theory, apply similar principles to generic medicines. Each stakeholder in the value chain (e.g., starting from funder/donors, pharmaceutical companies-generic and originator, medicines regulators, medicines procurement authorities, prescribers, dispensers, end users) bears some of the financial and reputational consequences of certain risks. We argue that behaviors are “misaligned” all along the pharmaceutical value chain, particularly when incentives and disincentives exist between adjacent stakeholders, as illustrated by misalignments among physicians and pharmacists with regard to wholesale, retail and consumer prices. As another example, quality regulators may, or may not, have an incentive to expedite regulatory approval of generics if they need to invest in more capacity or resources to do that [5]. Generic manufacturers need to be assured of higher volumes if they reduce wholesale prices.

Experience from high-income countries suggests that aligning the interests of different users and consumers of generics are necessary when selecting policy options. These options include: prescribing by generic name, generic substitution, financial incentives for pharmacy and medicines outlet personnel to sell low price generic medicines and continued education of consumers about generic medicines. However, many of these existing policies require insurance schemes to provide incentives to agents such as physicians or pharmacies to prescribe and dispense generic medicines.

## Conclusions

What policies should be given priority to promote generic medicines use in countries that do not have insurance systems that coverage a large proportion of the population? What policies can incentivize medicine dispensers to provide generic medicines in the absence of insurance schemes? Unfortunately, there is a paucity of relevant impact evaluations of pro-generic policies in LMICs [6]. For instance, various countries in the Asia Pacific region are attempting to co-ordinate implementation of generic medicines policies including procurement, re-imbursement, retail price controls, reference pricing and alignment of financial incentives among prescribers, dispensers and consumers to support the uptake of low-priced generic medicines. Evaluating these policies will be important to create evidence that other countries can use to implement policies in their local settings.

## Competing interests

The authors declare that they have no competing interests.

## Authors’ contributions

WK drafted the commentary, VW revised it; both authors approved the final version.
